# Tailoring of AlAs/InAs/GaAs QDs Nanostructures via Capping Growth Rate

**DOI:** 10.3390/nano12142504

**Published:** 2022-07-21

**Authors:** Nazaret Ruiz, Daniel Fernandez, Esperanza Luna, Lazar Stanojević, Teresa Ben, Sara Flores, Verónica Braza, Alejandro Gallego-Carro, Guillermo Bárcena-González, Andres Yañez, José María Ulloa, David González

**Affiliations:** 1 University Research Institute on Electron Microscopy & Materials (IMEYMAT), Universidad de Cádiz, 11510 Cádiz, Spain; nazaret.ruiz@uca.es (N.R.); teresa.ben@uca.es (T.B.); sara.flores@uca.es (S.F.); veronica.braza@uca.es (V.B.); david.gonzalez@uca.es (D.G.); 2 Paul-Drude-Institut für Festkörperelektronik, Leibniz-Institut im Forschungsverbund Berlin e.V., Hausvogteiplatz, 10117 Berlin, Germany; luna@pdi-berlin.de; 3 Institute for Systems Based on Optoelectronics and Microtechnology (ISOM), Universidad Politécnica de Madrid, 28040 Madrid, Spain; lazar@isom.upm.es (L.S.); alejandro.gallego@alumnos.upm.es (A.G.-C.); jmulloa@isom.upm.es (J.M.U.); 4 Department of Computer Science and Engineering, University of Cadiz, 11510 Cádiz, Spain; guillermo.barcena@uca.es (G.B.-G.); andres.yanez@uca.es (A.Y.)

**Keywords:** InAs QDs, AlAs capping, STEM, growth rate control

## Abstract

The use of thin AlA capping layers (CLs) on InAs quantum dots (QDs) has recently received considerable attention due to improved photovoltaic performance in QD solar cells. However, there is little data on the structural changes that occur during capping and their relation to different growth conditions. In this work, we studied the effect of AlA capping growth rate (CGR) on the structural features of InAs QDs in terms of shape, size, density, and average content. As will be shown, there are notable differences in the characteristics of the QDs upon changing CGR. The Al distribution analysis in the CL around the QDs was revealed to be the key. On the one hand, for the lowest CGR, Al has a homogeneous distribution over the entire surface, but there is a large thickening of the CL on the sides of the QD. As a result, the QDs are lower, lenticular in shape, but richer in In. On the other hand, for the higher CGRs, Al accumulates preferentially around the QD but with a more uniform thickness, resulting in taller QDs, which progressively adopt a truncated pyramidal shape. Surprisingly, intermediate CGRs do not improve either of these behaviors, resulting in less enriched QDs.

## 1. Introduction

Self-assembled intermediate band solar cells (IBSCs) have received special attention as an alternative to improve the current gain from the solar spectrum in single-junction solar cells, while maintaining a high open-circuit voltage (*V_oc_*) [1]. Quantum dot (QD) technology with confined electron levels has been proposed to experimentally study the operating principles of the IBSC concept [2]. Most QD-IBSC prototypes were manufactured based on the InAs/GaAs QDs system and grown by molecular beam epitaxy (MBE) using the Stranski-Krastanov mode [3,4,5,6]. InAs/GaAs QDSCs, while exhibiting considerable increase of the short-circuit current density, (*J_sc_*), result in an excessive reduction of the *V_oc_* causing poorer efficiency, as compared to their GaAs references [7,8]. The main reason adduced for the observed low performance is the presence of the wetting layer (WL) in between the QDs that generates continuous energy levels between the conduction band (CB) and the closely packed confined energy levels of holes inside the valence band (VB) [9,10]. This reduction in bandgap results in rapid capture of photoelectrons from the CB (GaAs) to the localized states in QDs, degrading the final efficiency. Therefore, the efficiency in IBSCs is strongly limited by the large phase volume of WL that diminishes the *V_oc_* [11]. Recently, the use of thin AlAs capping layers (CLs) has shown significant improvements in the *V_oc_* in InAs QDSCs, and this outcome was justified by a supposed elimination of the WL region [12,13]. However, in our previous works studying the effect of the CL thickness between one and ten monolayers (MLs) of AlAs, we have observed a substantial reduction of the WL for thicknesses of AlAs CL, but not a complete removal [14]. On the one hand, for the thinner AlAs CLs, the Al distribution around the QDs is non-uniform, tending to accumulate on the QD flanks at the expense of the apex, which is well below the nominal design [15]. On the other hand, for the thicker CL thicknesses, the CL is rather uniform, but QD populations split into a bimodal distribution with smaller lenticular QDs cohabiting with bigger truncated pyramids plastically relaxed.

In addition to the CL thickness, other parameters in the CL growth conditions could provide a tool to achieve a final structure with ideal functional properties, such as chemical nature, temperature, fluxes, growth rate, etc. [16,17,18]. In our case, it is not interesting to dilute the Al content using AlGaAs CLs, since even with the deposition of ultrathin layers of pure AlAs, the real Al contents observed in the layer are much lower than the nominal ones [14]. Regarding the CL growth temperature, almost all QD growers prefer to use the same temperature for CL as that used in QDs. The capping growth rate (CGR) is a not too explored tool that could strongly modify the final structure [19,20]. In general, lower CGR results in a decrease of both QD height and In content [21,22], showing a transition from taller pyramids to flatter lens-shaped QDs [23], but there are notable exceptions [18,24]. Anyway, there are no studies up to now on the changes of Al distribution around the InAs QDs with the CGR.

In this work, the impact of the AlAs CGR on the structural properties of the InAs/GaAs QDs system at the nanometer level has been studied using Scanning Transmission Electron Microscopy (STEM)-related techniques. On the one hand, it has been found that the population of QDs is strongly affected by changes in the CGR in terms of morphology, size, composition, and density. On the other hand, the distribution of Al, in both the WL region and around QDs, also varies with CGR. As will be shown, both results are related and do not lead to structural changes in the system in a stepwise manner.

## 2. Materials and Methods

**Figure 1:**
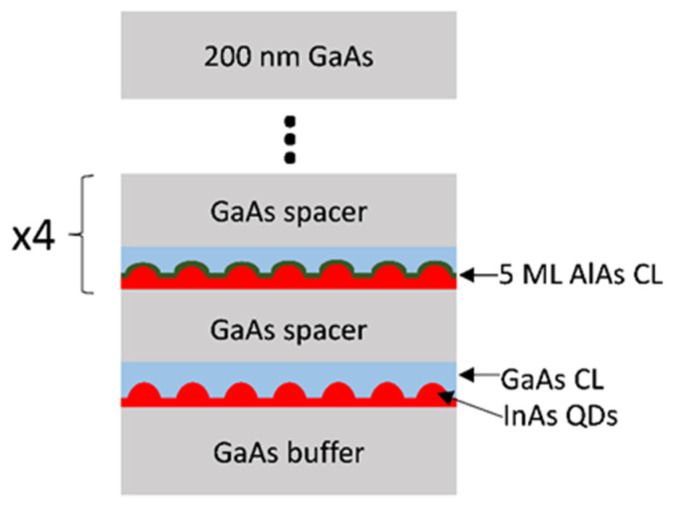
Scheme of the sample structure. The growth rate of the AlAs CLs increased in each layer, 0.25, 0.5, 0.75 and 1 ML/s, respectively.

The sample was grown by solid-source molecular beam epitaxy (MBE) on a Si-doped (100) n+ GaAs substrate. First, a 700 nm thick n-GaAs buffer layer was grown under As4 stabilized conditions. Over this, five different sequences of QD + CL, separated by 50 nm of GaAs grown at 610 °C, were sequentially deposited. Each QD layer was grown by depositing 2.7 MLs of InAs at a substrate temperature of 460 °C and a growth rate of 0.04 ML/s. The first CL consisted of 35 MLs of GaAs grown at 1 ML/s and 480 °C. The other 4 CLs were made of 5 MLs of AlAs, using different growth rates (0.25, 0.5, 0.75, and 1 ML/s, respectively) plus 30 MLs of GaAs, all grown at the same temperature of 480 °C. Each QD layer is hereafter referred to as CL#, where # is the growth rate of the CL of AlAs except for the CL without AlAs, which we call CL0. Finally, all the structure was covered by 200 nm of GaAs (see Figure 1). The further overgrowth had no significant influence on the shape of the dots, since the thermal activation energy prevented diffusion within the covered bulk material below 610 °C [25]. Simultaneous recording of Low Angle and High Angle Annular Dark Field (LAADF and HAADF, respectively) images and Energy-dispersive X-ray spectra (EDX) with ChemiSTEM^®^ (FEI Europe B. V., Eindhoven, The Netherlands) technology was performed in a double-aberration corrected FEI Titan Cubed ^3^ Themis operated at 200 kV and processed using Velox^®^ software. (Velox software Version: 2.12.1.37) Electron Energy Loss Spectra (EELS) registered in the same microscope to thickness map plotting were computed by Digital Micrograph^®^ software (Version: 3.43.3213.0). The required long acquisition times of EDX maps of individual QDs at high magnification may result in a loss of the spatial alignment of the spectral maps series due to drift phenomena in the TEM sample handler. To eliminate the possible drift problems, first, the Velox drift corrector was used in situ. Second, the spectra map series were post-processed, using the HAADF and LAADF images series acquired simultaneously as a reference, by an alignment algorithm. The algorithm is divided into 3 stages. In the first stage, the first image of the HAADF image series is used to perform a global alignment of the series to obtain an average image. In a second step, a local alignment is settled in each image regarding the average image. In the final stage, the drift measurements obtained from the alignment of the HAADF image series were applied to the EDX spectral map series. Both alignments were performed using normalized cross-correlation, together with the Vandewalle algorithm [26], for the correction of possible rotations.

## 3. Results

### 3.1. Structural Analysis of InAs QDs

In general, Diffraction Contrast (DC) TEM imaging using Dark Field (DF) g002 conditions is one of the most used methodologies for characterizing the morphology in III−V nanostructures, such as QDs, because of its simplicity and its sensitivity to compositional changes. However, this imaging technique presents great difficulties in the interpretation of the intensity contrast in the case of AlAs/InAs/GaAs QDs. The intensity has a complex dependence on the position, shape, and size of the 3D nanostructures, the thickness of the TEM sample, and the Al and In contents [15,27,28], so another imaging technique is needed. In principle, imaging methodologies based on ADF STEM are less sensitive to sample thickness [29,30] variations, so we tested them. Our proofs using compositional EDX maps for comparison showed that there were difficulties in intensity interpretation using HAADF images [15]. The intensity of HAADF images depends on the atomic number Z. In and Al atoms have opposite Z contrast contribution concerning Ga ones, so the contribution of In and Al to the column intensity may cancel each other out. Fortunately, the additional strain contribution to the intensity of LAADF images meant that, for TEM sample thicknesses below 100 nm, our QDs and the In-rich wetting layer were clearly distinguished in LAADF images as brighter contrast regions compared to the GaAs matrix, while the Al-rich CLs appear darker. The structural characteristics of the AlAs/InAs QDs, such as population, shape, and size were analyzed by using LAADF images.

**Figure 2:**
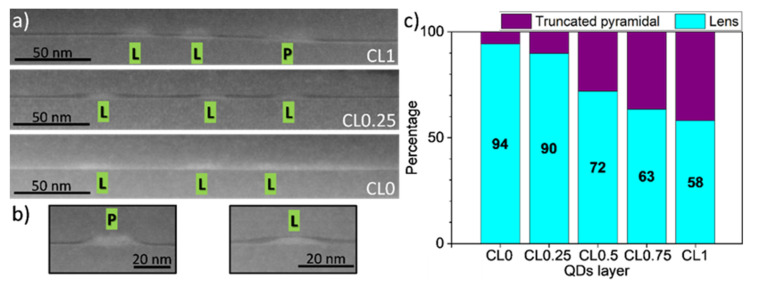
(**a**) LAADF images of the layers CL1 (top), CL0.25 (middle) and CL0 (bottom). Lens and pyramidal-shaped dots are marked with L and P, respectively. The Al-rich CLs appear darker. (**b**) Zoom of a truncated pyramidal (left) and lenticular (right) QD capped with AlAs. (**c**) The proportion of lenticular and pyramidal QDs in the QD population for the different QDs layers.

Figure 2a shows 3 cross-sectional LAADF STEM images taken along the [110] axis that corresponded to layers CL1 (top), CL0.25 (middle), and CL0 (bottom). In a first view, QDs presented different geometries and dimensions with different AlAs covering degrees. Focusing firstly on their shape, QDs might be classified as truncated pyramid QDs [31,32] (Figure 2b, left) and lenticular QDs [33,34] (Figure 2b, right). 

More than 50 QDs from each QD layer were classified attending to the geometries and the results are presented in Figure 2c. The shape was analyzed by enlarging the image of each QD and applying various color filters to decide which group it fell into. It is worth clarifying that these are not closed groups, as there is a transition from pyramidal to lenticular shape. Therefore, some QDs were challenging to classify, and they were not included in the statistics. As displayed in the bar graph, the vast majority of QDs without AlAs CL (layer CL0) showed a lenticular shape while only a small proportion of them presented the truncated pyramidal shape (<6%). This ratio hardly changed at the lowest AlAs CGR (CL0.25 layer), but gradually rose as the CGR became higher. Thus, for the highest AlAs CGR, CL1, there was almost parity between truncated pyramids (42%) and lenses (58%). Therefore, increasing the CGR led to better preservation of the original pyramid shape.

**Figure 3:**
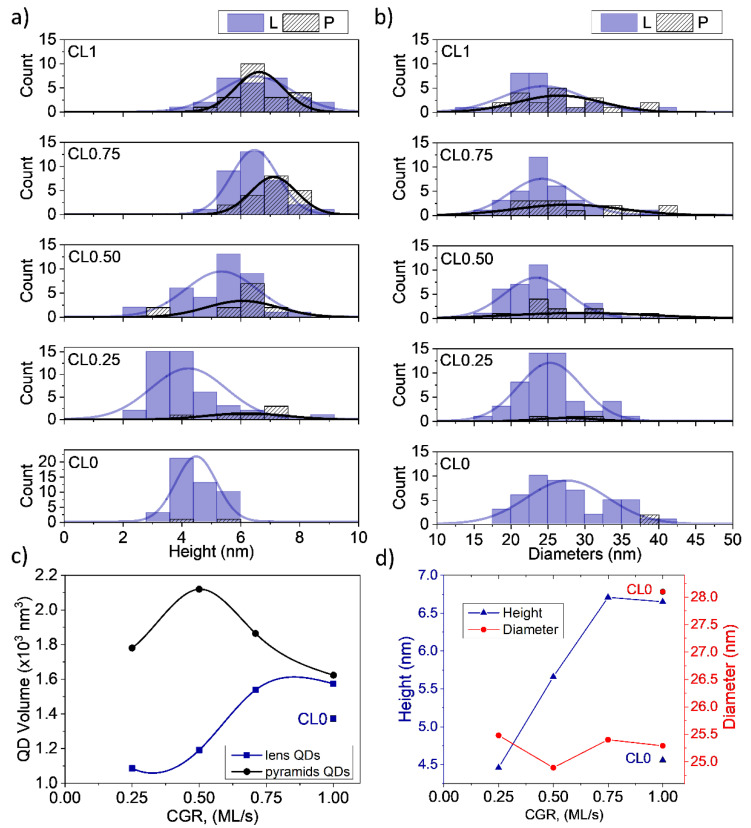
Histograms of (**a**) QD height and (**b**) base diameter distributions for lens and truncated pyramidal geometries of the different QD layers. The differences in the distribution between the two geometries become smaller as the CGR increased. Arrows show the position of the mean values of lenticular (red) and pyramidal (black) QDs. (**c**) The volume of QDs for lenticular and truncated pyramidal-shaped QDs versus CGR. Both geometries show similar average volume for the fastest CGR. (**d**) Average height and diameter of QDs versus CGR. The sample without CL of AlAs (CL0) is not connected by lines.

Focusing on the QD sizes, the heights, h, and base diameters, B, were obtained from LAADF images using the criteria of the intensity contrast around InAs QD established by comparison with EDX maps. Figure 3a,b displays the histograms of these parameters for each QD layer considering the different geometry together with their normal distribution curves. Remarkably, changes in the AlAs CGR influenced the dispersion of the QD size distribution. Thus, the histogram of QD heights in the CL0 layer had the lowest standard deviation compared to its counterparts with AlAs CL, although it improved with higher CGRs. This behavior is completely reversed in the case of QD diameter, where CL0 had the largest standard deviation, but its counterparts with AlAs CL improved with lower CGRs. It seemed as if the improvement in the dispersion of one parameter, e.g., the height, produced the worsening of the other parameter, the base diameter, being impossible to improve both at the same time.

Figure 3c is a plot of the average QD volume as a function of the CGR making a distinction on the QD shape. In general, the truncated pyramid shape was more common for larger QDs, while the lens shape was preferable for smaller QD volumes [35]. As can be seen, the few truncated pyramid-shaped QDs were twice as large as the lenticular ones for the lowest CGR. For CGRs above 0.5 ML/s, the volume difference narrowed down to almost null in the case of 1 ML/s. What was more, QD volumes in CL1 were over those of the CL0 one. These results were in line with a lower general decomposition of QDs at higher CGRs and AlAs capping, and a higher tendency to preserve the original shape and size of the QDs before capping.

As the size parameters converged when the population of the different shapes was balanced, it was reasonable to represent the overall mean height and diameter of each QD layer as in Figure 3c, without making the distinction of QD shape. It has been widely reported that AlAs reduce QD dissolution during capping, so taller QDs should be expected with AlAs CL compared to ones with GaAs capping [12,15]. Still, this was not the case for QDs covered by AlAs at the lowest CGR, CL0.25, where the QD height average (4.5 nm) was like that obtained in the CL0 layer. A progressive increase of QD heights occurred at higher CGRs reaching a plain stabilization in the range of 0.75–1 ML/s of about 6.7 nm. Hence, the deposition rate of the AlAs CL seemed to be a crucial parameter to control the QD heights. Regarding the diameter base, the introduction of AlAs induced a small reduction from 28 nm to 25 nm in the lateral dimensions, which seemed not to be affected by changing the CGR. Therefore, the aspect ratio, h/B, experienced a slight increase from 0.17 in the CL0 layer to 0.26 in the CL1 layer. The values were within the typical range observed during the capping of InAs QDs by the usual capping layers [33].

**Figure 4:**
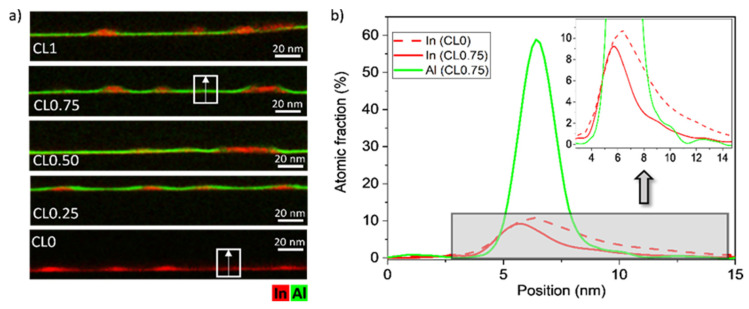
(**a**) Elemental EDX maps of In and Al for the different QD layers. (**b**) Average compositional profiles of In (red) and Al (green) along the growth direction in the CL/WL regions of the QD layers CL0.75 and CL0. All the compositional profiles of the QD layers with CL of AlAs in the regions between dots are remarkably similar. The inset corresponds to a zoom of the gray region.

As we reviewed in the introduction, the amount of InAs in the WL is an indicator of the degree of QD decomposition and has direct implications for the quantum efficiency in QDSCs. Figure 4a shows elemental EDX maps of the five QD layers where red and green hue intensities correspond to In and Al contents, respectively. The CLs of AlAs were remarkably similar and homogeneous in the regions between dots, though irregularities could be seen around the QDs. In addition, WL regions of InGaAs between QDs seemed to blur after AlAs capping. However, multichannel images could be misinterpreted if both elements were in the same position. To solve that, average compositional profiles for In and Al in regions between QDs were taken along the growth direction. Notably, all the compositional profiles of the QD layers with CL of AlAs in the regions between dots were similar, so Figure 4b only plots the case of CL0.75 as an example, together with the QD layer without AlAs, CL0. The compositional profiles in CL0.75 evidence the presence of Al almost from the beginning that cohabited with In, forming a quaternary alloy as we have observed previously [14]. When comparing both QD layers, the introduction of AlAs capping provoked a reduction by over 34% of the area under the In profiles concerning the CL0 layer. Therefore, the WL was thinner with the presence of AlAs CLs due to an important constraint in the QD decomposition, but it was almost independent of the CGR.

**Figure 5:**
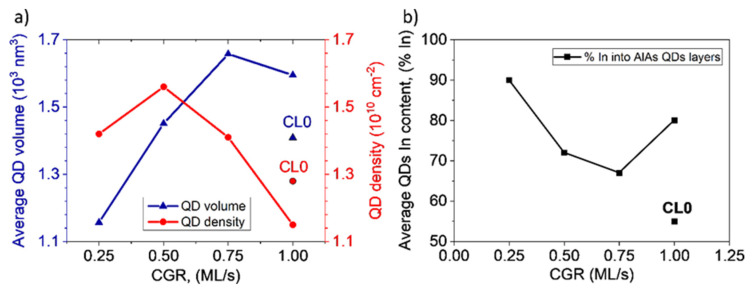
(**a**) Average QD volume (left axis) and superficial density (right axis) versus CGR (**b**) Average In content obtained for the different layers. Better protection of dots is seen for extreme AlAs CGR.

The effect of the AlAs CGR on QD densities was also investigated. For this purpose, simultaneous low-loss EELS maps and LAADF images were acquired in STEM mode in the same areas. LAADF images allow counting the number of QDs in each layer while EELS maps offer an accurate measure of the sample thickness in each position by using the log-ratio method [36,37]. The density values ranged from 1.1 × 10^10^ cm^−2^ to 1.6 × 10^10^ cm^−2^ so the QDs density was not notably influenced by the CGR. The values of QDs density are compared in Figure 5a to the average QD volume, calculated taking into consideration the geometry. As can be seen, a slight decrease in the QD density happened at higher CGRs at the expense of an increase in QD volume. This inverse relationship between average QD volume and superficial density was recently described in other systems [15,21]. During capping, the best strategies to avoid decomposition of QDs lead to preferential removal of smaller QDs, discouraging decomposition in larger QDs, resulting in a net reduction in QD density and an increase in mean volume [38].

It is possible to calculate the average composition of the QDs following the procedure proposed by Joyce et al. [39]. The total amount of material deposited, 2.7 ML of InAs, is distributed between the WL and the QDs. The area under the In profile in the WL regions permits estimating the total amount of In in the WL region, so its difference with the total content deposited is the amount of In in the QD population [40]. Now, the average In contents within the QDs for each layer could be calculated considering QD densities and the average QD volume. Figure 5b shows the results of the average In contents into the QDs for the layers with AlAs CL (black line) together with the CL0 reference layer. The values obtained are only descriptive of the general tendency since the accumulated error of the measurements could be up to 10%. Two important conclusions can be drawn from this graph. First, all QDs covered by AlAs were In-richer than those without AlAs. Second, the relationship between volumes and In contents was not linear. On the one side, the QDs capped with AlAs at the lowest CGR had the smallest sizes but the highest In contents. On the other side, the QDs in the layer with the highest CGR presented the largest volume, but not the highest In content. The largest QDs did not always have the richest In contents [21]. Layers with intermediate rates had the lowest In contents.

### 3.2. Analysis of AlAs CL around the QD

**Figure 6:**
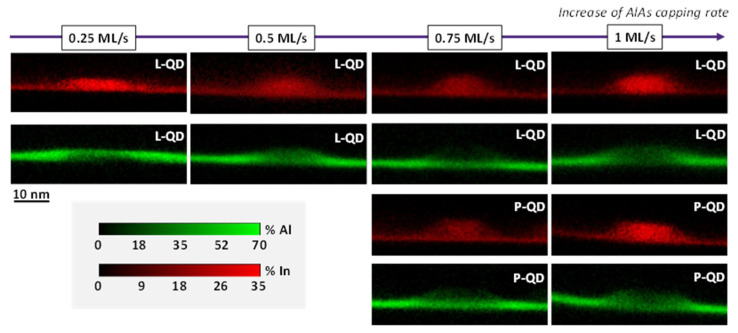
In (red) and Al (green) EDX maps of representative lens-shaped QDs (the top two rows) for the QD layers with CL of AlAs. We have also included examples of truncated pyramidal-shaped QDs (the bottom two rows) when the proportion of pyramids is significant (CL0.75 and CL1 layers).

Our results showed that CGR affected the QD characteristics, such as shape, size, density, and composition, but not the WL ones. Surprisingly, QDs grown with the extreme CGRs were In-richer, compared to those grown at intermediate ones, and this fact could not be related to a change in WL characteristics [15,21,40]. We suspect that the changes in the QDs structure might be due to variations of the CL structure around the QD at different CGRs. Figure 6 shows in the top two rows high magnification EDX maps for representative lens-shaped QDs of each layer, where green and red hue colors correspond to Al and In contents, respectively. Additionally, examples of truncated pyramidal QDs are also included in the bottom 2 rows for the layers CL0.75 and CL1, since the percentage of this geometry was noticeable. Regarding the In distribution, QDs seemed to follow the trends seen in Figure 5a, where the extreme CGRs gave rise to higher In contents in the QDs. If we focus on the Al maps, the coverage around the dots changed for the different CGRs, where lenticular QDs achieved smoother capping than pyramidal QDs, especially for the lowest CGR.

**Figure 7:**
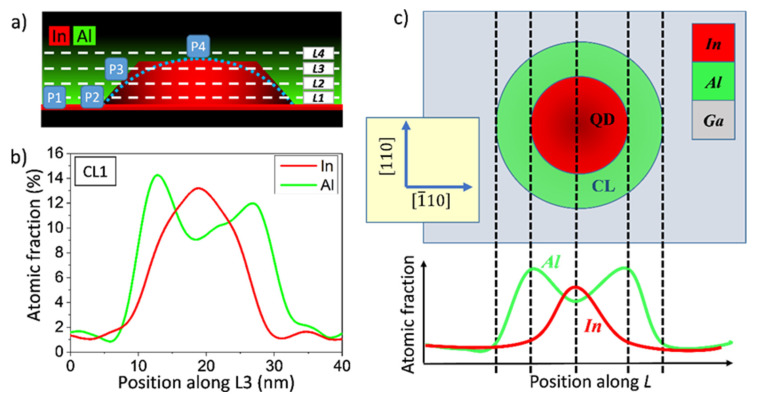
(**a**) Scheme of a QD capped by an AlAs layer. L1 to L4 are directions parallel to the growth plane where the compositional profiles are taken. P1 to P4 are the positions for punctual measurements. (**b**) Experimental compositional profiles along the line L3 for a QD in the CL1 layer. (**c**) Plan view of a slide of the QD capped with AlAs and its correspondence with the theoretical composition profiles for In and Al.

However, visual inspection of the compositional EDX maps did not provide straightforward quantitative information on the CL changes around the dots. With the goal of obtaining measurements of the thickness and content at different heights of the CL around QDs, 4 profiles were taken parallel to the interface along the directions labeled in Figure 7a, from the base of the QD (*L1*) to the upper region above the QD (*L4*). An example of the obtained profiles is shown in Figure 7b. A single peak in the In profile surrounded by a double peak in the Al profile could be observed. For better clarification, Figure 7c shows a plan view slice of the system containing one of these directions above, and below, the theoretical Al and In profiles. The In profile always showed a single maximum while the Al profiles outlined a symmetric curve consisting of a valley surrounded by two peaks. The base of the In peak and of the twin peaks in the Al profiles corresponded to the diameter of the QD and the CL at that height, respectively [15]. In addition, for each profile, the diameter of the InAs QD was also the distance between the peak’s maxima in the Al profile.

**Figure 8:**
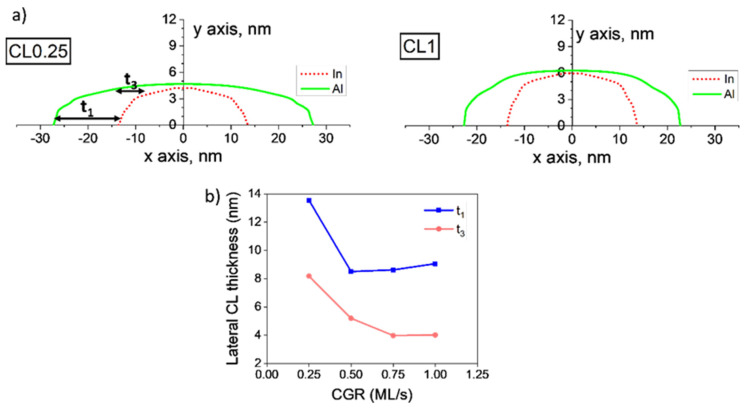
(**a**) Average contours of the QD and CL for layers CL0.25 and CL1. Lateral thicknesses of the CL at the base and close to the apex are named *t*1 and *t*3, respectively. (**b**) Lateral thicknesses *t1* and *t3* of the CL versus the CGR.

According to this procedure, diameters of the QD and CL at different heights were measured for several QDs in each layer. For simplicity, Figure 8a shows average contours only for the layers with the extreme CGRs (CL0.25 and CL1 layers) since the layers CL0.5 and CL0.75 exhibited intermediate features. Figure 8b displays the lateral thicknesses of the CL, *t*_1_, and *t*_3_, obtained using the profiles *L1* and *L3*, respectively for the 4 CGRs. At the base, *t*_1_, QDs with the lowest CGR (CL0.25) showed a huge lateral thickening of the CL with respect to all the QDs capped at higher CGRs, which showed similar values. The thickness of the CL decreased as we moved up the QD (*t*_1_ is about double that of *t*_3_) but was always thicker than over the apex. In summary, Al atoms tended to accumulate on the lateral flanks of QDs, especially near the base, and there was a breakup effect in the case of the lowest CGR.

**Figure 9:**
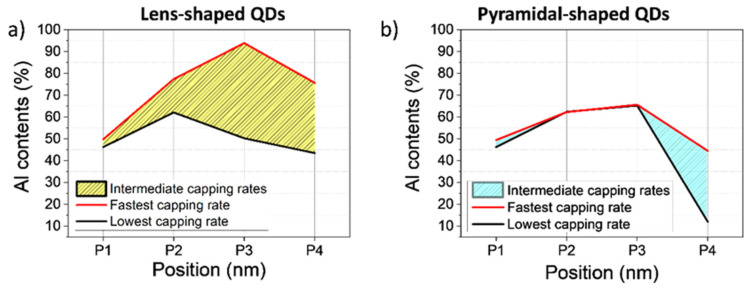
Al contents in the CL for lenticular (**a**) and truncated pyramid QDs (**b**) in the positions marked in Figure 7. (P1) corresponds to regions of the WL away from the QD, (P2) at the QD edge, (P3) close to the QD top and (P4) above the QD.

The different degrees of coverage in terms of thicknesses ought to play a crucial role in the structural and functional changes of the QDs, not only in terms of thicknesses but also concerning Al content. To obtain a more complete picture of the capping structure in terms of Al composition, we selected 4 positions for the evaluation of the CL composition, from a position away from the QD (*P1*), at the QD edge (*P2*), near the top (*P3*) and above the QD (*P4*) (Figure 7a). The procedure to estimate the Al contents in the CL in each position took into account the sample thickness, and the dot geometry is detailed in Ref. [15]. Figure 9 displays the averaged Al contents for these 4 positions obtained from representative QDs with lenticular (left) and truncated pyramidal geometries (right). The black line corresponds to the values for the lowest CGR (CL0.25) and the red line for the highest CGR (CL1). The values measured from QDs with intermediate CGRs were within this region. For lens-shaped QDs and the lowest CGR, the CL showed an almost homogeneous distribution with contents like that of the WL region, while accumulating strongly around the QD for the highest CGR, reaching up to ~90% Al content at the top of the QD (P3). If we focus on QDs with a truncated pyramidal shape, the Al content in the CL around the QD was remarkably similar for all CGRs, being different only at the apex. In this case, the apex was almost devoid of Al for the lowest CGR, with a progressive accumulation of Al on the apex for higher CGRs. The contents at the QD flanks were like those seen in the WL.

## 4. Discussion

**Figure 10:**
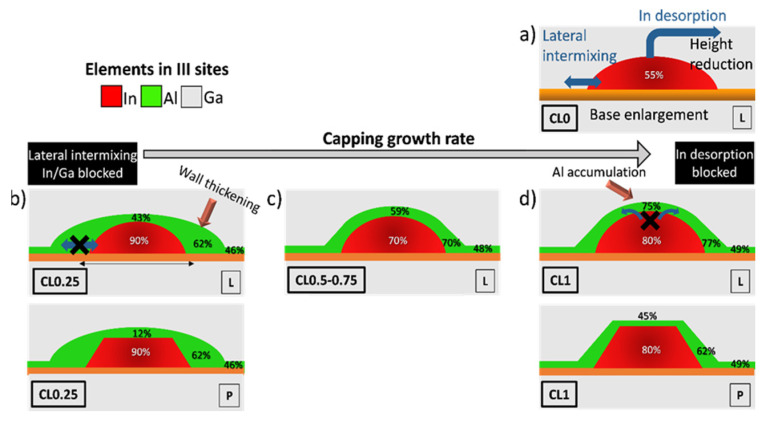
Schematic representation of the CGR effects in the GaAs/InAs/AlAs QD system for a (**a**) QD capped with GaAs (CL0), (**b**) covered by AlAs at 0.25 ML/s (CL0.25), (**c**) 0.5–0.75 ML/s and (**d**) 1 ML/s (CL1). “L” and “P” at the bottom right in each picture designate the lens and truncated pyramidal geometries. Average compositions for the QDs and the CL are indicated.

To understand the morphological evolution of QDs during the capping process it is necessary to know which principles govern their epitaxial growth. The dot shape and size are equilibrium properties, and stable islands form when the total energy related to facet formation and elastic strain is reduced. For small QDs, the preservation of steeper facets implies an energetic cost, which is not yet counterbalanced by the energy gain of strain relief in the upper part of the island, hence resulting in QDs with lens geometry. For larger islands, a higher aspect ratio is found to be preferable due to more efficient strain relaxation in the steep part of the island [41]. In addition, during the capping process, the strain introduced by a GaAs CL in the QD can be relieved by an out-diffusion of In from the QD apex together with In/Ga intermixing in the laterals. Therefore, this causes a rapid decomposition of the QDs resulting in the following: (i) a reduction of the QD height where part of the dot material is transferred to the WL, and (ii), an enlargement of the QD diameter because of a Ga/In interchange that causes a dilution of the In content within the dot (Figure 10a) [33,42].

In the case of AlAs capping, the higher energy barriers for surface diffusion of Al (0.8 eV) [43] suggest that Al atoms are rather immobile, remaining close to the first surface adsorption place, compared to Ga (0.62 eV) [44] or In (0.4 eV) atoms [45], the latter having higher mobility. Consequently, a high degree of QD coating is to be expected from the initial stages of AlAs deposition. However, the accumulation of Al on the QD increases the stress at the apex and the contrary occurs at the edges, so there is a tendency for Al to migrate from the apex to the QD flanks due to better lattice matching [46]. Thus, although the surface diffusion length of Al is very short, during the AlAs capping of QDs at the lowest CGR (CL0.25), Al atoms have enough time to migrate from the apex to the QD flanks, so a huge thickening occurs in the CL of the QD laterals at the expense of the CL apex, which is impoverished (Figure 10b). In contrast, at the highest growth rate (CL1), the CL around the dots has the highest Al content, much higher than that seen in the regions between the dots. Certainly, Al atoms show a predilection to have a first adsorption site that is preferentially above the QDs with respect to the regions between dots. In general, atomic kink sites on the island facets are preferential sites for atomic adsorption, which helps to reduce the barrier for an adatom to chemically bond to the epitaxy. In addition, the shorter surface diffusion time in this high-speed condition has a freezing effect to preserve this configuration and this explains the elevated content of Al around the QDs (Figure 10d).

Besides all this, the changes of the CGR not only affect the Al distribution around the QD but also the morphology of the QDs. It is reported [22] that an extreme growth rate (2 ML/s) using a CL of GaAs almost preserves the initial geometry of the unburied QDs, yielding tallest truncated pyramidal QDs [22]. Lower capping rates (≤1 ML/s) lead to a QD shape evolution from pyramidal shapes to a lens-shaped QD. Certainly, in the QD layer grown with a GaAs CL at a CGR of 1 ML/s (CL0), the prevailing geometry was the lenticular shape with a small proportion of truncated pyramidal shape (<6%). At the same CGR, the presence of AlAs (CL1) weakened the QD decomposition processes by reducing both the In atom desorption from the apex [12] and the degree of lateral In–Ga intermixing during growth [47] (Figure 10d). However, the strength of these effects depended strongly on the amount of Al covering the apex and lateral flanks. On the one hand, at the lowest CGR with AlAs (CL0.25), there was a remarkable accumulation of Al on the QD flanks leading to a huge increase in the lateral CL thickness (*t_3_*~8 nm) while the Al content over the apex reduced (Figure 10b). Therefore, the lateral In/Ga mixing was highly blocked, so the dilution of In content was exceptionally low. The longer times for surface diffusion resulted in higher migration of Al from the apex that favored desorption of In, so the apex erosion was high. Thus, there was a predominance of shorter lenticular QDs with high In content. On the other hand, at the highest CGR (CL1) Al was accumulated around the QD, reaching up to Al content above the QD of about 75% with a thinner thickness (Figure 10d). Now, desorption of In from the apex was deterred, resulting in higher QDs and, in many cases keeping the pyramidal shape (42% (CL1) versus 6% (CL0)). The lower lateral thickness of the CL was compensated by the high Al content, resulting in a tiny dilution of the QD. At intermediate CGRs, a gradual decrease in Al content around the dot occurred concerning CL1, resulting in a decreasing inhibitory effect on apex desorption and a reduction in QD height. However, lateral thickening hardly increased. Therefore, both effects combined produced a higher dilution of In within the QD due to higher lateral intermixing compared to the extreme cases (Figure 10c). Our results suggest that controlling the Al distribution, through proper manipulation of the overlayer growth rate, can be an effective tool to improve the structure of self-organized InAs QDs, and can help to tailor their physical properties to any specific requirements of the device applications.

## 5. Conclusions

In summary, our results prove that the CGR of AlAs affects both the QD features (volume, density, shape, size, and composition) and the Al distribution around the QDs, but not the WL features, which have similar features at all rates. Analyzing QDs, we saw the presence of lens and truncated pyramidal geometries where the proportion of pyramidal QDs increased from the lowest (6%) to the highest (42%) CGR. In addition, an increase in QDs heights without changes in diameters occurred by increasing CGRs. Remarkably, we found that the QDs covered by AlAs at the lowest and highest CGRs had the higher In contents. The changes in the Al distribution around the QDs with the CGR were the main reason for this behavior. On the one hand, the lowest CGR results in a CL with a homogeneous content over the epitaxial surface, although thickening strongly on the sides of the QDs. This higher thickness hinders lateral mixing in the QDs, which would result in a lower dilution of the In content during capping, with respect to capping with GaAs. On the other hand, the highest CGR results in a preferential accumulation of Al atoms in the QDs that blocks the desorption of In from the apex during capping, achieving the tallest QDs. However, the shorter surface diffusion time prevents lateral thickening resulting in a thinner CL. Intermediate CGRs do not improve either of these behaviors, resulting in less enriched QDs.

## Data Availability

Not applicable.
